# Decoding the regulatory code: O-GlcNAcylation in epithelial-mesenchymal transition (EMT)

**DOI:** 10.1016/j.jbc.2026.111265

**Published:** 2026-02-06

**Authors:** Shisheng Zhou, Wenhui Lou, Zijun Wei, Teng Wang, Yang Li, Qijie Zhao, Fan Zhang, Ye Nie, Hui Qian, Zhiwei Xu

**Affiliations:** 1 Jiangsu Key Laboratory of Medical Science and Laboratory Medicine, School of Medicine, Jiangsu University, Zhenjiang, Jiangsu, China; 2 Clinical Laboratory of Xuanwu Hospital, Capital Medical University, Beijing, China

**Keywords:** cancer metastasis, EMT, O-GlcNAcylation, post-translational modification, therapeutic targeting

## Abstract

O-linked N-acetylglucosamine (O-GlcNAc) is a monosaccharide modification occurring on serine or threonine residues of most eukaryotic proteins. Only two enzymes, O-GlcNAc transferase and O-GlcNAc hydrolase, regulate the dynamic flux of O-GlcNAc modification, rendering it extremely responsive to nutrition and stress conditions. O-GlcNAcylation stands at the center of epithelial-mesenchymal transition (EMT), sensing nutrient and stress signals to direct the transcriptional and signaling programs that enable phenotypic plasticity, thereby establishing its fundamental role in fibrosis and tumor metastasis. EMT is an essential biological event that confers mesenchymal characteristics to epithelial cells, characterized by the suppression of E-cadherin, a key epithelial adhesion molecule, and the overexpression of N-cadherin, a mesenchymal cadherin that promotes motility, or Vimentin, a mesenchymal intermediate filament protein. This review covers recent insights on the multiple canonical and non-canonical roles of O-GlcNAc, presenting O-GlcNAc cycling as a significant post-translational mechanism involved in various aspects of EMT. Furthermore, we systematically examine the functional connections between O-GlcNAcylation and EMT, focusing on identifying key O-GlcNAcylated proteins that regulate EMT and evaluating the relative contributions of transcriptional and post-translational mechanisms mediated by this modification. A comprehensive understanding of the intricate molecular circuitry governing the interplay between O-GlcNAcylation and EMT will deepen our mechanistic insights into cellular plasticity and offer novel therapeutic avenues for combating metastasis and other EMT-associated pathologies.

Post-translational modification (PTM) is a dynamic and vital modification that can be added or removed from the peptide chain by specialized enzymes, thereby enriching its chemical sequence and carrying information content (1, 2, 3). Growing evidence shows that PTMs play a crucial role in numerous physiological activities, including the regulation of protein activity and metabolism (4), as well as the maintenance of protein stability (5). Since it was first discovered by Hart’s laboratory in 1984 (6), O-GlcNAcylation has been demonstrated to intersect with a wide spectrum of physiological and pathological processes, from transcription and metabolism to cellular stress response. O-GlcNAcylation involves the addition of a single O-GlcNAc moiety to the serine and threonine residues of proteins, and it is solely regulated by a pair of key enzymes, namely O-GlcNAc transferase (OGT) and O-GlcNAc hydrolase (OGA) (7). Consequently, O-GlcNAcylation does not pertain to complex glycans or branching structures (8). Glucose enters the body, and approximately 5% of it flows into the hexosamine biosynthetic pathway (HBP), which is regulated by key enzymes such as glutamine fructose-6-phosphate amidotransferase 1 (GFAT1) and phosphoglucosamine mutase 3, ultimately generating uridine diphosphate N-Acetylglucosamine (UDP-GlcNAc) (9). As a glycosylation donor substrate, UDP-GlcNAc is involved in the intracellular O-GlcNAc modification process catalyzed by OGT and OGA (10, 11). O-GlcNAcylation, which requires resources like glucose, glutamine, and glucosamine, is crucial for nutrient sensing and the maintenance of physiological processes, exhibiting distinct spatiotemporal control (11) (Fig. 1).

**Figure 1:**
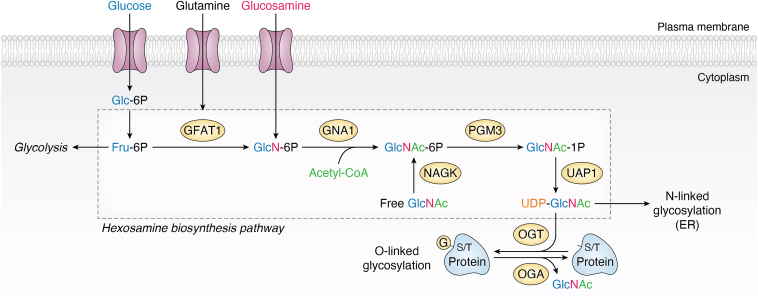
**Nutrients provide substrates for O-GlcNAcylation *via* the HBP pathway.** Three nutrients, Glucose, Glutamine, and Glucosamine, were synergistically taken up by GLUT and SLC from the extracellular milieu to the cytoplasm. While most glucose fuels glycolysis and glycogen synthesis, approximately 5% enters the HBP. Fructose-6-phosphate—a key intermediate derived from glucose catabolism—undergoes conversion to glucosamine-6-phosphate through a reaction catalyzed by glutamine fructose-6-phosphate amidotransferase, the rate-limiting enzyme in this pathway. Subsequently, glucosamine-6-phosphate is subject to acetylation and uridylation catalyzed by enzymes such as Glucosamine-phosphate N-acetyltransferase 1 and PGM3, ultimately leading to the formation of UDP-GlcNAc. UDP-GlcNAc is then used for N-linked and O-linked glycosylation. This metabolite serves as the essential donor substrate for protein serine/threonine sites’ O-GlcNAcylation, a dynamic post-translational modification cycle regulated by OGT (*adds*) and OGA (*removes*). Free GlcNAc can be efficiently recycled into HBP through the action of N-Acetylglucosamine Kinase. HBP, hexosamine biosynthetic pathway; OGA, O-GlcNAc hydrolase; OGT, O-GlcNAc transferase.

O-GlcNAcylation, a dynamic protein modification that reacts to cellular cues, is essential both in physiology and pathology (12, 13). Physiologically, it serves as a key nutrient-sensitive sentinel, modulating essential processes including gene expression (transcription and translation), protein homeostasis, signal transduction, and metabolic flux. It directly modulates essential circadian rhythm proteins to sustain daily biological processes and is crucial for mitochondrial flexibility to energy requirements, including facilitating brown fat thermogenesis under cold stress (12, 14). Pathologically, dysregulated O-GlcNAcylation contributes to disease progression. In neurodegenerative diseases like Alzheimer's (15), aberrant modifications disrupt protein folding and transport, aggravating cellular damage and cognitive deterioration (16, 17, 18). In cancer, elevated O-GlcNAcylation facilitates carcinogenesis by hyperactivating oncogenic signaling pathways, promoting unregulated cell proliferation, augmenting stress response adaptation, and eventually aiding cancer cell survival and development within the tumor microenvironment (19).

Epithelial-mesenchymal transition (EMT) describes a process of molecular remodeling and phenotypic change from polarized and immotile epithelial cells to mesenchymal cells (20), a phenomenon initially documented by Elizabeth Hay in the early 1980s (21). Notably, the plasticity of EMT is underscored by its reversibility through mesenchymal–epithelial transition (MET), a process marked by the reassembly of intercellular junctions and recovery of apical–basal polarity (22). Nonetheless, EMT that frequently transpires only partially in tumor cells is referred to as partial or hybrid EMT (23, 24). Integrating multiple cellular processes, EMT is central to cellular reprogramming and phenotypic plasticity, playing pivotal roles in diverse contexts from embryonic development to organ fibrosis and cancer metastasis (20, 22). The EMT is thought to transpire at the invasive front of epithelial tumors, facilitating the migration and invasion of cancer cells into surrounding tissues and blood vessels, which is the central theme of our discussion (25).

Despite the ambiguity surrounding the specific classifications of EMT programs, the EMT process is categorized into three groups based on the biological context (26). Type I EMT occurs throughout embryonic development characterized by critical cycles of EMT and MET regulated by conserved transcription factors (27, 28), enabling cell differentiation and organ morphogenesis (29, 30). Type II EMT participates in tissue repair, including wound healing and regeneration, as well as organ fibrosis (31, 32). Although it produces fibroblasts essential for tissue repair following injury, sustained activation during chronic inflammation leads to pathological fibrosis and organ damage (33). Type III EMT is linked to cancer growth and metastasis, facilitating the detachment, invasion, and dissemination of carcinoma cells (34). The EMT process involves a multitude of molecular players, among which transcription factors (TFs) play a central regulatory role in its initiation and progression. Key players include snail family transcriptional repressor 1 SNAIL1 (SNAI1), snail family transcriptional repressor 2 SLUG (SNAI2), zinc finger E-Box binding homeobox factors ZEB1 and ZEB2, as well as basic helix-loop-helix factors TWIST1 (TWIST) and TWIST2 (25). Subsequently, master regulatory EMT-TFs orchestrate phenotypic switching by repressing epithelial genes (*e.g.*, *CDH1, OCLN, CLDN1, KRT18*) while activating mesenchymal genes (*e.g.*, *CDH2, VIM, FN1*). These transcription factors and essential proteins interact and influence one another to establish a complex and intricate molecular regulatory network that regulates the EMT process, significantly impacting cellular destiny and functional transformation (Fig. 2).

**Figure 2:**
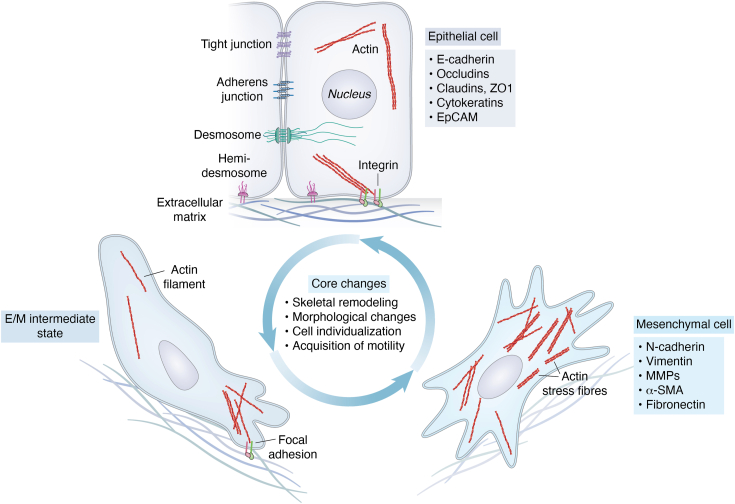
**Cells undergo the EMT process and core alterations.** Cells exhibiting both epithelial-like and mesenchymal-like features possess distinct characteristics that enable them to adapt to different environments. Epithelial cells adhere to each other *via* tight junctions, adherens junctions, and desmosomes, and are tethered to the basement membrane *via* hemidesmosomes. These cells express a variety of molecules associated with the epithelial state (listed in the *gray box*) to maintain cellular polarity. In contrast, mesenchymal-like cells shed epithelial characteristics and establish front-rear polarity, driven by actin stress fiber formation and cytoskeletal reorganization. This reorganization activates the expression of genes associated with the mesenchymal state (listed in *blue boxes*). Cells gain mobility and invasiveness during EMT. EMT is a reversible process in which mesenchymal cells are restored to epithelial cells by MET. The transition state between these two phenotypes is termed the intermediate state. Both EMT and MET occur during normal development as well as in cancer progression. EMT, epithelial-mesenchymal transition; MET, mesenchymal–epithelial transition.

O-GlcNAcylation significantly influences the onset of EMT, facilitating Type III EMT-mediated metastasis. This PTM significantly modifies essential EMT components, including E-cadherin, N-cadherin, and Snail, consequently influencing their stability and subcellular location. The disruption of cell polarity, frequently driven by O-GlcNAcylation-mediated destabilization of adherens junctions and apical-basal determinants, instigates the acquisition of invasive phenotypic traits—including cytoskeletal remodeling, matrix degradation, and amoeboid morphology—which collectively enable metastatic dissemination.

The mechanism by which O-GlcNAcylation affects EMT is not simply through cumulative modification levels, but relies on the highly dynamic cycling on and off of OGT/OGA (35, 36). Cells maintain O-GlcNAc levels by regulating the activity, expression, and subcellular localization of these enzymes, while integrating metabolic pathways (such as the HBP pathway) and the supply of substrate precursors (37). In pathological conditions such as a tumor, increased glucose uptake and activation of the HBP pathway due to the Warburg effect disrupt this balance, driving the cycle towards the 'on' state. Importantly, this overall level change is accompanied by specific modifications of certain substrates (such as EMT-related transcription factors), and through crosstalk with modifications like phosphorylation, forms a complex regulatory network that ultimately precisely drives the EMT process.

## Crosstalk between O-GlcNAcylation and EMT

### O-GlcNAcylation globally affects EMT

EMT is a tightly regulated transformation process wherein cells experience metabolic reprogramming to redistribute energy sources, including glucose (38, 39). O-GlcNAcylation serves as an essential chemical switch, enabling cells to detect and respond to fluctuations in food availability and external signals. Variations in O-GlcNAcylation significantly influence EMT (40, 41). As a key metabolite of the HBP, UDP-GlcNAc serves as the substrate for O-GlcNAcylation, which directly modulates the functions of EMT-related transcription factors and signaling molecules and facilitates the metabolic reprogramming essential for the EMT process (42, 43, 44).

The mechanistic details of O-GlcNAcylation-mediated EMT regulation can be elucidated using diverse experimental approaches. To begin, pharmacological modulation of O-GlcNAcylation levels *in vivo* robustly modulates the EMT process. The OGT inhibitor OSMI-1 suppresses cigarette smoke-induced EMT by downregulating SMAD family member 4 (SMAD4) expression (45). The same trend was found in endometrial cancer cells, hyper-O-GlcNAcylation in Thiamet-G-treated cells enhanced EMT protein expression and cytoskeletal reorganization. Conversely, lower O-GlcNAcylation also enhances EMT-related gene expression without affecting cytoskeletal reorganization (46).

**Figure 3:**
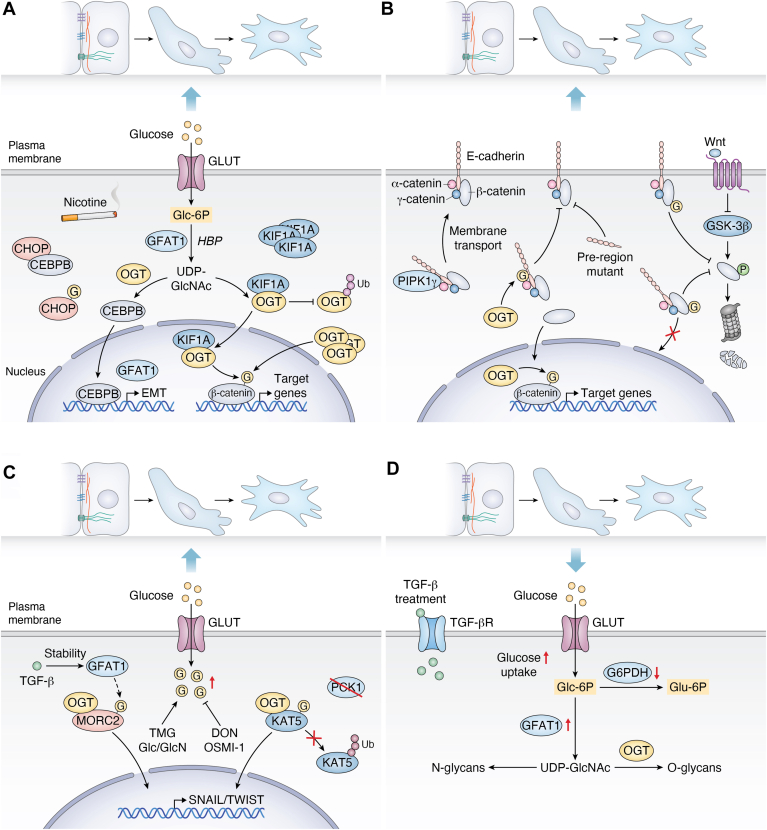
**The phenotypic outcomes of the O-GlcNAcylation-EMT interplay.***A*, O-GlcNAcylation globally affects EMT. Nicotine-stimulated overexpression of O-GlcNAcylation promotes EMT activation. O-GlcNAcylation of CHOP prevents its inhibition of C/EBPB, allowing C/EBPB to enter the nucleus, thereby further increasing the transcription of GFAT1 and forming a positive feedback loop (*left*). Overexpression of kinesin-like protein binds to OGT, preventing its degradation and thereby increasing OGT stability and nuclear accumulation. OGT in the nucleus can increase O-GlcNAcylation of β-catenin, thereby promoting EMT. Additionally, overexpression of OGT itself also promotes O-GlcNAcylation of β-catenin in the nucleus (*right*). *B*, core molecules undergoing O-GlcNAcylation promote EMT. O-GlcNAcylation of E-cadherin inhibits its translocation to the cytosol, specifically by inhibiting its binding to PIPK1γ, but not its binding to β-catenin, α-catenin, and γ-catenin; furthermore, mutations in the pre-region inhibited the translocation of E-cad to the cytosol. O-GlcNAcylation of β-catenin synergizes with the Wnt signaling pathway to inhibit ubiquitin-proteasomal degradation mediated by GSK-3β and to inhibit its entry into the nucleus. β-catenin without O-GlcNAc modification can be translocated to the nucleus and modified by OGT there, thereby promoting the expression of EMT-related genes. *C*, noncore molecules undergoing O-GlcNAcylation also promote EMT. TGF-β induces OGT-mediated O-GlcNAcylation of MORC family CW-type zinc finger 2 by promoting the stabilization of GFAT1, which increases the transcriptional level of Snail essential for EMT activation, thereby promoting the migration and invasion of breast cancer cells (*left*). Phosphoenolpyruvate carboxykinase 1 deletion promotes OGT-mediated O-GlcNAcylation of KAT5, thereby inhibiting its degradation; the stabilized KAT5 can then induce the expression of EMT-related genes (*right*). *D*, EMT inversely affects O-GlcNAcylation. TGF-β induces the development of EMT. During this process, the level of glucose uptake increases, while the activity of PPP and the key glycolytic enzyme G6PDH decreases, and the level of GFAT1 increases. The increased HBP flux mediates abnormal cellular O-GlcNAc levels. TMG: Thiamet-G, an OGA inhibitor; Glc/GlcN: Glucose/Glucosamine; DON: 6-Diazo-5-oxo-L-norleucine, a GFAT1 inhibitor; OSMI-1: an OGT inhibitor; PPP, Pentose phosphate pathway. EMT, epithelial-mesenchymal transition; HBP, hexosamine biosynthetic pathway; MET, mesenchymal–epithelial transition; OGA, O-GlcNAc hydrolase; OGT, O-GlcNAc transferase.

It is suggested that any fluctuation in O-GlcNAcylation homeostasis contributes to significant changes in EMT. Previous findings indicated the pharmacological intervention of OGT/OGA in EMT, and ongoing investigations are contributing to our expanding understanding of the EMT programs through genetic intervention of HBP enzymes. However, the precise regulatory mechanisms governing these changes are not fully understood. Nicotine stimulation increased GFAT1 transcription in breast cancer cells, thereby elevating O-GlcNAc levels. Moreover, heightened O-GlcNAcylation enhanced EMT signaling, ultimately fostering an invasive phenotype (47) (Fig. 3*A*). O-GlcNAcylation, which functions as a "sensor" of cellular metabolic status, is an integral regulator of EMT. It directly modulates the function of EMT-related transcription factors and signaling molecules. Critically, O-GlcNAcylation drives the metabolic reprogramming essential for EMT. Both pharmacological and genetic interventions aimed at O-GlcNAc homeostasis primarily regulate the overall O-GlcNAcylation level. However, understanding O-GlcNAc homeostasis requires assessing not only the 'overall level' but also its high 'site specificity'. Therefore, global O-GlcNAc homeostasis does not mean that every site on every protein is properly modified. Changes in O-GlcNAc levels of site specificity could modulate EMT protein expression, cytoskeletal reorganization, and cell invasion across diverse models, highlighting its potential role in EMT.

### Core molecules in EMT undergo O-GlcNAcylation

As a fundamental process of cell fate plasticity, EMT serves a vital role in embryonic development, tissue healing, and cancer metastasis. Despite a comprehensive understanding of EMT across diverse domains, including epigenetic regulation, tumor microenvironment, and metabolic reprogramming, a key question persists. The mechanism by which cells detect microenvironmental metabolic variations (*e.g.*, glucose, hypoxia) and translate them into molecular signals for EMT initiation remains enigmatic. Numerous studies indicate that key EMT regulators, such as Snail and β-catenin, can undergo aberrant hyper-O-GlcNAcylation. This elevated modification promotes the acquisition of invasive capabilities in tumor cells by downregulating the expression of epithelial markers (*e.g.*, E-cadherin) and enhancing the production of mesenchymal phenotypic signals. Engagement of E-cadherin by its major component of adherens complex—including the pivotal β-catenin—connects its cytosolic domain with the actin cytoskeleton and maintains lateral epithelial connections between neighboring epithelial cells (48). Moreover, engagement of canonical EMT inducer Snail by transcriptionally repressing E-cadherin expression (49). Direct nuclear interaction of the transcription factor Snail with β-catenin, thereby enhances β-catenin’s transcriptional activity (50). These discoveries elucidate a clear molecular connection between metabolic stress and EMT processes, while also offering a novel approach to targeting the “glucose metabolism-related modification” axis for intervention in tumor metastasis (Fig. 3*B*).

#### β-catenin

Expansive signaling pathways are involved in EMT, including TGF-β, Notch, and Wnt/β-catenin (20, 26, 29). The Wnt/β-catenin pathway occupies a key node in the complex regulatory network of metazoans (51). β-catenin, a dual-function protein, operates at the nexus of Wnt signaling and cell adhesion (52, 53, 54). Multiple post-translational modifications have been identified in β-catenin, including the discovery of O-GlcNAc on β-catenin in 2014, which plays a crucial role in maintaining protein stability. The O-GlcNAc modification of β-catenin in T41 inhibits the proteasomal degradation of β-catenin by directly competing with phosphorylation, thereby stabilizing β-catenin (55). Together with this study, O-GlcNAcylation not only positively regulates the stability of β-catenin but also affects its localization in adhesive junctions. The O-GlcNAcylation of β-catenin at Ser23 is crucial for maintaining mucosal integrity, enhancing its association with α-catenin, and supporting its localization at the plasma membrane. The GFP-S23G mutant was shown to be ineffective in preventing β-catenin aggregation following OGA inhibition by PUGNAc (56). O-GlcNAcylation of β-catenin restricts its nuclear translocation, thereby impairing its interaction with T-cell factor/lymphoid enhancer factor and resulting in reduced transcriptional activation of Wnt target genes (57). Moreover, O-GlcNAcylation modulates β-catenin production and activity, directly influencing cellular proliferation and invasion (58, 59). The O-GlcNAc modification of β-catenin has been demonstrated in protein stability and localization at the plasma membrane, yet whether this modification modulates these characteristics during the EMT process remains a crucial unanswered question. Collectively, these findings establish O-GlcNAcylation as a master regulator that controls both the stability and adhesive function of β-catenin, positioning it as a critical integrator of signaling and cell adhesion during EMT (Fig. 3*B*).

#### E-cadherin

As a key component of the classical cadherin superfamily, E-cadherin serves as the principal cell adhesion molecule in epithelial tissues (60). E-cadherin, a key calcium-dependent homophilic adhesion molecule, maintains epithelial integrity by mediating intercellular adhesion, while its downregulation or functional impairment is closely linked to EMT (61). Similar to β-catenin, E-cadherin can also be modified by O-GlcNAcylation (62). This modification, taking place in the cytoplasm, disrupts the transport of E-cadherin to the cell membrane surface, ultimately resulting in impaired intercellular adhesion capacity. Specifically, core EMT-TFs (SNAIL, TWIST, and ZEB1) directly repress E-cadherin transcription, whereas O-GlcNAcylation post-translationally modulates its activity (63). E-cadherin was initially identified with O-GlcNAcylation by soluble wheat germ agglutinin-pull down (sWGA-pull down), although no specific site was identified (64). O-GlcNAcylation impairs E-cadherin function through two complementary mechanisms. First, disrupted anterograde trafficking sequesters newly synthesized E-cadherin into non-functional intracellular pools, reducing surface localization and compromising intercellular adhesion by preventing junctional cytoskeletal assembly. Second, O-GlcNAcylation directly disrupts E-cadherin–p120-catenin binding, destabilizing the cadherin-catenin complex. This dual impairment drives adherens junction disassembly and promotes EMT progression. The O-GlcNAcylation of E-cadherin is also involved in regulating cell adhesion during the EMT process (63). In MCF-7, elevated O-GlcNAcylation can reduce intercellular adhesion and promote the transformation of epithelial cells into mesenchymal cells. Compared with the classical way of down-regulating E-cadherin through the transcriptional level, O-GlcNAcylation regulates E-cadherin function more rapidly (64). Therefore, as a nutrient-sensitive modulator *in vivo*, O-GlcNAcylation can flexibly and dynamically adjust the function of E-cadherin according to environmental changes, and may fulfill a critical dynamic regulatory function in the initiation, progression, and reversal of EMT. While the functional impact of O-GlcNAcylation on E-cadherin is well-established, key knowledge gaps persist regarding its role during EMT. These include incompletely defined context-dependent regulatory mechanisms upstream of E-cadherin’s O-GlcNAcylation, as well as the underexplored crosstalk with other post-translational modifications, such as phosphorylation, governing the multidimensional spatiotemporal control of E-cadherin function and EMT progression.

#### Snail1

Snail1 functions as a principal transcriptional repressor that binds to the E-box elements in the E-cadherin promoter, thereby repressing its transcription, while concurrently promoting the expression of mesenchymal markers (65, 66). By orchestrating this genetic reprogramming, it drives EMT, facilitating cell motility and metastatic dissemination (67). Genome-wide RNA-seq and protein interaction analyses reveal that Snail1, a key EMT transcription factor, directly interacts with OGT across multiple genomic loci, validated by co-immunoprecipitation and RT-PCR assays. This finding identified Snail1 as a key substrate of OGT, which modulates transcriptional activity and protein level. Consequently, this OGT-Snail regulatory axis governs downstream biological processes, including cell adhesion, migration, and differentiation (68). Indeed, Snail possesses an O-GlcNAc modification at Ser112. This modification stabilizes Snail1 by competing with phosphorylation at the same or adjacent residues, thereby preventing its targeting for proteasomal degradation (69). More precisely, the glucose content was tightly correlated with the O-GlcNAc level, and elevated glucose levels promoted the O-GlcNAcylation of Snail1. Interestingly, it is reasonable to state that the inhibition of O-GlcNAc levels in Snails regulates the transcription of EMT genes. Therefore, it is of great interest to dissect the Snail1 O-GlcNAc status of various types of tissues affected by fibrosis in the chronic hyperglycemic state. The OGT-mediated Snail1 O-GlcNAcylation establishes a direct coupling between O-GlcNAc signaling and transcriptional reprogramming during EMT. Concurrently, glucose-dependent O-GlcNAcylation of Snail1 at specific residues (*e.g.*, Ser112) stabilizes the transcription factor, generating a self-reinforcing feed-forward loop. This integrated mechanism precisely coordinates nutrient sensing with EMT execution, thereby controlling cell adhesion, migration, and differentiation programs.

#### Other molecules

Beyond β-catenin and E-cadherin, EMT progression is orchestrated by O-GlcNAcylation of additional molecular players. Emerging studies reveal that dynamic O-GlcNAcylation critically regulates key EMT drivers, including Vimentin, SMAD4, ZEB1, and Cofilin. O-GlcNAc modification of vimentin is required for the maintenance of intermediate filament morphology and cell migration capacity (70). O-GlcNAcylation at its head Ser49 site is essential for homologous interactions of waveform proteins. O-GlcNAcylation at Thr63 is both sufficient and necessary for SMAD4 stabilization by blocking GSK-3β-mediated proteasomal degradation, substantially prolonging its half-life. In contrast, this defective modification attenuates the luciferase reporter gene activity of the TGF-β-responsive SMAD-binding element, suggesting that the O-GlcNAc modification stabilizes SMAD4 by regulating the TGF-β/SMAD signaling pathway (71). ZEB1 is similarly a core protein in EMT, but its O-GlcNAcylation plays additional roles in non-EMT progression. Specifically, O-GlcNAcylation of ZEB1 at the Ser555 locus under high-glucose conditions enhances its stability and nuclear translocation, induces transcriptional activity of lipogenesis-related genes, and ultimately leads to lipid peroxidation-dependent ferroptosis in mesenchymal pancreatic cancer cells (72).

O-GlcNAcylation acts as a critical "regulatory switch," masterfully coordinating the function of key proteins to profoundly influence EMT progression. It serves as a vital molecular link, integrating the regulation of HBP enzymes—such as GFAT1 and OGT—with the transcriptional reprogramming required for EMT and metastasis. This establishes a pioneering perspective: when cells sense these pathophysiological stresses, O-GlcNAc modification of core EMT regulators is enhanced, which can stabilize proteins like E-cadherin under specific contexts and facilitate cellular adaptation. Future research should focus on elucidating how O-GlcNAcylation participates in the entire EMT cascade and interacts with other post-translational modifications, as well as its cell-type-specific functions in pathology.

### Non-core molecules in EMT undergo O-GlcNAcylation

While certain classical EMT molecules, such as β-catenin and E-cadherin directly influence the EMT process *via* O-GlcNAcylation, recent in-depth studies have revealed that numerous traditional molecules, not classified as "core regulators of EMT," can also indirectly inhibit or collaboratively promote EMT progression through this dynamic modification. These non-core molecules are extensively present in essential pathways, encompassing metabolic control, cytoskeletal dynamics, signal transduction networks, and microenvironmental sensing. Their O-GlcNAcylation changes can facilitate the intricate EMT by altering energy metabolism, enhancing mechanical signaling, or modifying cell-extracellular matrix interactions. This extensive modification occurring across numerous molecules forms a "non-core molecular network", uncovering the intricate regulatory web of EMT.

Non-core EMT molecules can orchestrate EMT processes by regulating molecules within the EMT through O-GlcNAcylation. Kinesin-like protein (KIF1A) was found to be highly expressed in neuroendocrine prostate cancer, and its knockdown impaired neuroendocrine features, including EMT, while overexpression promoted these processes. Overexpression of KIF1A promotes nuclear accumulation of OGT and O-GlcNAcylation of intranuclear β-catenin, which is crucial for invasive tumor growth (73) (Fig. 3*C*). In addition to altered molecules within the core regulators of EMT proteins *via* O-GlcNAc, the O-GlcNAcylation can directly occur on non-core EMT molecules, which reveals expanded regulations of the O-GlcNAcylation in EMT. Lysine acetyltransferases (KATs), which mediate the attachment of Lysine acetylation to proteins, is one of the major protein post-translational modifications (74). KAT5 is critically associated with cancer cell proliferation and metastasis, although a number of other regulations have been proposed (75). The presence of O-GlcNAc on KAT5 was confirmed upon treatment with Thiamet-G (the OGA inhibitor). Further research reveals that the S119A mutant decreased O-GlcNAc levels of KAT5 and increased c-Myc degradation. OGT-mediated O-GlcNAcylation of KAT5 promoted by knockdown of phosphoenolpyruvate carboxykinase 1 (PCK1) resulted in activated transcription of TWIST1 and diminished the expression of E-cadherin, while enhancing the expression of N-cadherin. In the PCK1-KO MHCC-97H cells, WT KAT5, but not the S119A mutant, leads to a marked increase in the EMT process. Notably, OGT mediates the core mechanism by which O-GlcNAcylation maintains KAT5 stability during EMT. In this study, they demonstrate that KAT5 stability is primarily maintained through O-GlcNAcylation-mediated suppression of ubiquitin-dependent degradation, highlighting an important mechanism by which O-GlcNAcylation regulates protein stability (76). However, KAT5 is subject to multiple post-translational modifications (77, 78, 79), and whether O-GlcNAcylation influences other regulatory modifications—such as phosphorylation or SUMOylation—remains an open question.

The active research in the O-GlcNAc field has led to emerging information for extensive studies of O-GlcNAc in non-core EMT molecules. Previous research has also demonstrated that MORC family CW-type zinc finger 2 (MORC2) is O-GlcNAcylated at Thr556 (80). At the transcriptional level, O-GlcNAcylated MORC2 is required for SNAIL transcriptional activation. Similarly, O-GlcNAcylation of forkhead box A1 promotes its assembly with chromatin and can stimulate the expression of downstream adhesion-related genes. Although it does not directly affect EMT, its O-GlcNAc site-specific mutations can inhibit tumor cell metastasis (81). Hence, O-GlcNAcylation can fine-tune the EMT process according to the metabolic state of cells by dynamically regulating the O-GlcNAcylation of non-core molecules. These different examples implicate that the role of O-GlcNAc in EMT can rely on non-core EMT molecules driving the transition. Interestingly, the O-GlcNAcylation of different substrates among non-core proteins may result in many variations of the EMT program. It remains uncertain how O-GlcNAc specifically controls non-core EMT molecules or whether there is a common switch that ultimately activates this transition. Finally, additional studies are needed to comprehensively map O-GlcNAc modification sites on non-core EMT-related proteins.

Overall, modifying non-core EMT molecules through O-GlcNAcylation intricately regulates the EMT process both transcriptionally and post-translationally. Deciphering the regulatory interplay between O-GlcNAcylation and non-canonical EMT molecules is crucial for elucidating its pathophysiological roles and facilitating translational advances in EMT-associated research, diagnosis, and therapeutic development.

## EMT inversely affects O-GlcNAcylation

Although converging evidence has elucidated O-GlcNAcylation-mediated regulation of EMT through discrete pathways, the integrated regulatory network bridging these processes remains inadequately characterized. In recent years, it has been reported that the changes in O-GlcNAcylation also occur during EMT (82). It has been proposed that EMT regulates protein O-GlcNAcylation, a process that involves two central mechanisms. The initial molecular mechanism identified to demonstrate the relevance of EMT-mediated O-GlcNAcylation regulation is the activation of the HBP. Lung cancer, a particularly fatal malignancy, enhances glucose absorption and channels it into the HBP pathway during the occurrence of EMT in A549 cells. The activation of HBP subsequently results in an elevation of aberrant cellular O-GlcNAcylation. Although the majority of glucose influx is channeled toward glycolysis and the pentose phosphate pathway, the increased glucose uptake observed during EMT in these cells is primarily diverted into the HBP. This metabolic shift is driven by a corresponding increase in the protein expression and enzymatic activity of GFAT1, the rate-limiting enzyme of the HBP. This resulting increase subsequently leads to a progressive upregulation of UDP-GlcNAc levels (73). In contrast to the O-GlcNAcylation-mediated EMT process, this research showed that exposure of cells to TGF-*β* induced O-GlcNAc and represented increased OGT levels. The second central mechanism identified to demonstrate the importance of O-GlcNAcylation driven by EMT is the transcriptional regulation of OGT and OGA. Twist is a key transcription factor that triggers EMT, a process conferring increased resistance to chemotherapy (83, 84). Most importantly, TWIST1 stability is strikingly maintained largely due to the O-GlcNAc modification and TWIST1 subsequently regulates OGT expression, suggesting a potential feedback loop between the EMT transcription factor TWIST1 and OGT. The mechanistic study largely supports the roles of EMT in O-GlcNAc with respect to this reciprocal regulation. These two mechanisms—increasing the UDP-GlcNAc (HBP flux *via* GFAT1) and transcriptionally increasing the crucial enzyme (OGT)—act in concert to promote overall O-GlcNAcylation during the EMT process.

Certainly, it is acceptable to assert that the regulation of O-GlcNAcylation by EMT is precise and multilayered: 1) Changes in the course of EMT lead to metabolic reprogramming, such as increased glucose flux, and the increased glucose provides substrate support for O-GlcNAc. 2) EMT-related signaling pathways such as TGF-β and PI3K/Akt may regulate the activity and expression of OGT/OGA in the activated state. For instance, PI3K can act as a protein kinase phosphorylating the T985 of OGT and significantly enhancing the catalytic activity of OGT (85). 3) EMT-TFs such as SNAIL and TWIST may bind to the promoter region of OGT/OGA and potentially regulate the transcriptional level of OGT/OGA (86). 4) EMT is usually accompanied by stress responses such as endoplasmic reticulum (ER) stress and oxidative stress. ER stress regulates O-GlcNAc through the unfolded protein response (UPR), and splicing-type X-box binding protein 1 is a conserved UPR signaling factor and highly active transcription factor. It has been demonstrated to enhance intracellular O-GlcNAcylation by directly upregulating the expression of HBP genes (87). Emerging evidence positions HBP as a putative master regulator of EMT (44, 88). By controlling substrate flux for O-GlcNAcylation—a potent modulator of EMT transcription factors and adhesion complexes—HBP activity may dynamically orchestrate epithelial plasticity across both developmental and pathological contexts (76, 89, 90). O-GlcNAcylation both regulates and is induced by EMT *via* metabolic reprogramming, forming a self-reinforcing cycle that propels metastasis (Fig. 3*D*).

## Mechanisms of O-GlcNAcylation in regulating EMT progression

### O-GlcNAcylation synergizes with phosphorylation to regulate EMT plasticity

**Figure 4:**
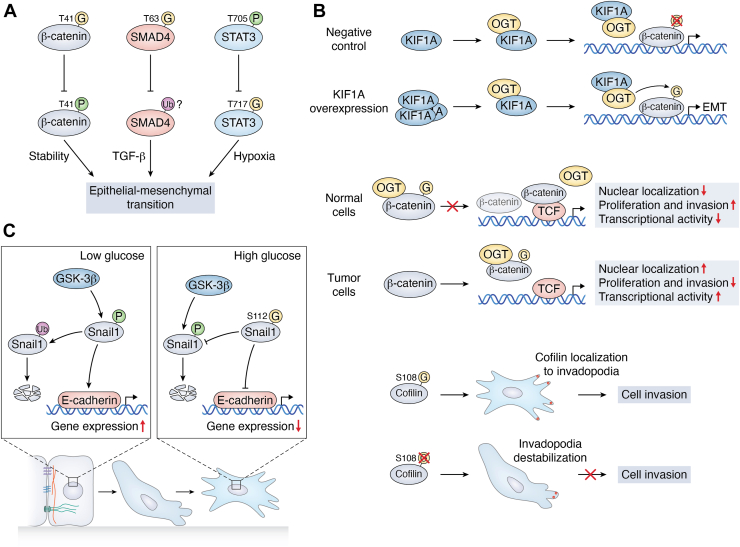
**Mechanisms of O-GlcNAcylation affect EMT.***A*, O-GlcNAcylation synergizes with phosphorylation to regulate EMT plasticity. O-GlcNAcylation of β-catenin at T41 inhibits phosphorylation at the same site; O-GlcNAcylation of SMAD4 at T63 inhibits its ubiquitination and degradation; and phosphorylation of STAT3 at T705 inhibits O-GlcNAcylation at T717, thereby contributing to the process of EMT. *B*, O-GlcNAcylation alters protein localization to affect EMT. Overexpression of kinesin-like protein promoted its binding to OGT to form a complex and induced the entry of OGT into the nucleus, and O-GlcNAcylation of β-catenin in the nucleus promoted the process of EMT; O-GlcNAcylation of cytoplasmic β-catenin inhibited its translocation to the nucleus. In normal cells, nuclear β-catenin combines with T cell factor to promote cell proliferation and invasion. In tumor cells, β-catenin can be transported to the nucleus, thereby increasing O-GlcNAc levels in the nucleus, which further inhibits its binding to T cell factor, thereby preventing cell proliferation and invasion. O-GlcNAcylation of cofilin can support its localization to invadopodia at membrane protrusions and promote breast cancer cell invasion. However, disrupted O-GlcNAc modification at Ser-108 of cofilin has been shown to destabilize invadopodia and impair cell invasion. NC/OE: Negative control/Overexpression. *C*, O-GlcNAcylation alters protein stability to affect the EMT process. Glucose availability determines Snail stability and E-cadherin expression: Low glucose activates GSK-3β–mediated Snail phosphorylation and degradation, relieving E-cadherin repression, while high glucose triggers Snail O-GlcNAcylation at Ser112, blocking GSK-3β phosphorylation and suppressing E-cadherin transcription. EMT, epithelial-mesenchymal transition.

PTMs represent a fundamental mechanism for controlling protein function and structure, with phosphorylation and ubiquitylation being classic examples (2, 91, 92). O-GlcNAcylation is another key reversible PTM that shares this mechanistic feature, primarily modifying serine and threonine residues similar to phosphorylation (8). Recent research has further strengthened the connection between O-GlcNAcylation and phosphorylation. Glucose-mediated O-GlcNAcylation of Casein kinase II at Ser347 regulates the assembly and dissociation of the COP9 Signalosome 2-Cullin-RING ligase 4 complexes by modulating the phosphorylation of COP9 Signalosome 2 at Ser21/Ser24 (93). O-GlcNAcylation at Ser112 stabilizes the EMT transcription factor Snail by blocking phosphodegron-mediated proteolysis. This post-translational switch potentiates Snail's repression of E-cadherin, initiating EMT (69). Unlike Snail, β-catenin has been most classically studied, as both of its two modifications occur at Thr41. Interestingly, due to its occurrence at the same site, O-GlcNAcylation and phosphorylation of Thr41 compete with other (55). Multiple modifiable sites within protein domains enable intricate crosstalk between O-GlcNAcylation and phosphorylation, orchestrating EMT through dynamic functional reprogramming. This regulation operates through two interconnected mechanisms: competition at shared sites, where both modifications target identical or adjacent residues on a single protein, enabling synergistic or antagonistic interplay through spatial proximity, and cross-protein coordination, where modifications on distinct proteins collectively orchestrate EMT progression (Fig. 4*A*). Together, these mechanisms exemplify how posttranslational modifications fine-tune cellular transformation *via* integrated spatial and hierarchical regulatory networks. It is possible that O-GlcNAcylation of protein A triggers the phosphorylation cascade reaction of protein B through changes in conformation or subcellular localization. To summarize, O-GlcNAcylation and phosphorylation interactions are mainly mediated through site competition, metamorphic regulation, and modulation of OGT/OGA itself. Through precise site competition and regulation of protein-protein interactions, O-GlcNAcylation is capable of modulating the expression of core EMT molecules, such as E-cadherin, consequently driving the invasion and metastasis of tumor cells.

### O-GlcNAcylation regulates molecular subcellular localization to alter the EMT process

Altered subcellular localization of key molecules during EMT is the central mechanism driving the process (94). Core EMT transcription factors, including SNAIL and ZEB, enter the nucleus to directly repress epithelial genes and activate mesenchymal transcriptional programs. E-cadherin restricts its transport to the surface of the cell membrane after being modified by O-GlcNAc (62). Dynamic changes in cellular localization play a crucial role in enabling precise spatiotemporal regulation, thereby facilitating transitions in cellular phenotypes. Many of these alterations in localization are reversible, thereby enhancing the flexibility in transitioning between EMT and MET (95). Notably, E-cadherin, a pivotal molecule in EMT, undergoes caspase-dependent O-GlcNAcylation in the cytoplasmic structural domain duringER stress-induced apoptosis. O-GlcNAcylation traps E-cadherin intracellularly, ablating its cell-surface adhesive function (62, 64). Moreover, non-core EMT molecules can also indirectly regulate O-GlcNAcylation levels of EMT molecules to promote the EMT process. Take KIF1A as an example. Although KIF1A localization minimally impacts EMT directly, it indirectly regulates EMT by stabilizing OGT and promoting its nuclear accumulation—enabling O-GlcNAcylation of nuclear β-catenin (73). However, O-GlcNAcylation drives tumor metastasis through protein mislocalization—a hallmark EMT phenotype. Cofilin, a key actin-severing protein, primarily drives cell motility by depolymerizing and severing existing actin filaments, thereby generating new barbed ends for rapid actin polymerization, which is essential for membrane protrusion and cell crawling (96, 97). The O-GlcNAcylation of Ser108 is crucial for cofilin to accurately localize at the front of breast cancer cells during three-dimensional (3D) cell invasion (98). Following the loss of this modification, the cellular invasive capacity is compromised. O-GlcNAcylation orchestrates EMT progression through spatiotemporal control of core regulators. By trapping E-cadherin intracellularly, it ablates epithelial adhesion while enabling nuclear translocation of EMT-TFs (SNAIL/ZEB) and β-catenin (*via* KIF1A-OGT axis). Site-specific modification (*e.g.*, cofilin-Ser108) directs actin-remodeling proteins to invasion fronts, while reversible localization switches permit phenotypic plasticity. This compartmentalized regulation establishes a dynamic control system where subcellular positioning dictates functional outcomes, linking metabolic signaling to metastatic competence (Fig. 4*B*).

### O-GlcNAcylation regulates protein stability to drive the EMT process

An important function of O-GlcNAcylation is maintaining protein stability by modulating chaperone interactions and inhibiting degradation pathways (99). O-GlcNAcylation governs protein stability primarily through three distinct mechanisms: the stabilization of EMT-related proteins, the regulation of diverse non-EMT-related proteins, and the maintenance of OGT/OGA stability.

The O-GlcNAc modification of EMT-related proteins has been shown to modulate protein stability through suppressing phosphorylation (69, 100). O-GlcNAcylation at Serine 112 stabilizes Snail1 by counteracting phosphorylation-mediated degradation, thereby potentiating its repressive activity and leading to suppression of E-cadherin transcription. Under hyperglycemic conditions, enhanced O-GlcNAc modification reinforces this regulatory axis, promoting Snail1-mediated E-cadherin silencing and instigating EMT (69) (Fig. 4*C*).

Some reports suggest that the stabilization of EMT-related proteins within the cell is dependent on O-GlcNAcylation, and other research has indicated that non-canonical EMT proteins promoting the EMT process of cancer cells display cancer metastasis associated with O-GlcNAcylation. Checkpoint kinase 2 (CHK2) plays a crucial role in the tumorigenesis of HCC. A research group has newly reported a mechanism by which O-GlcNAcylation regulates the stability of the CHK2 protein. O-GlcNAc levels at Thr378 of CHK2 increase dramatically in the absence of PCK1, thereby inhibiting ubiquitination to protect it from degradation. In contrast, Thr378 mutation compromises CHK2 stability and drives its proteasomal degradation (101).

Lastly, the stability of O-GlcNAc-modifying enzymes is essential for precisely orchestrating O-GlcNAcylation dynamics, thereby playing a pivotal role in shaping EMT processes. The ubiquitin-protein ligase E3 module N-recognition 5 (UBR5) plays a role in controlling the stability of OGA, thereby indirectly influencing the EMT process (35). UBR5 is markedly upregulated in gemcitabine-resistant pancreatic cancer cells and mediates this resistance by promoting O-GlcNAcylation-mediated EMT. OGA is a negative regulatory enzyme for O-GlcNAcylation; further studies confirmed that UBR5 is the E3 ubiquitin ligase of OGA. Through binding to OGA, UBR5 mediates a decrease in OGA stability, which in turn promotes O-GlcNAc levels and EMT activation. O-GlcNAcylation drives the EMT process and invasive phenotype by stabilizing pro-oncogenic proteins through the regulation of protein degradation. The mechanism of O-GlcNAcylation regulation of the stability of numerous molecules in EMT remains to be explored.

## Conclusions and perspectives

Since its discovery, pioneering work has cataloged the expanding landscape of O-GlcNAcylated proteins, enabling functional characterization of this PTMP (6). This review critically assesses the functional contribution of O-GlcNAcylation to the EMT process. Specifically, we address five critical, unresolved questions concerning its role in EMT pathogenesis: 1) the context-dependent regulation of EMT by O-GlcNAcylation; 2) identification of critical O-GlcNAcylated substrates that mediate EMT progression (Table 1); 3) the mechanistic crosstalk between O-GlcNAcylation and established EMT signaling pathways; 4) the reciprocal regulation of O-GlcNAcylation status by the EMT program; and 5) the maintenance or disruption of O-GlcNAc homeostasis during the EMT process.

**Table 1:** EMT-related molecules undergo O-GlcNAc modification

Core molecules	O-GlcNAcylation sites	Function	Ref.
β-catenin	T41/S23	Maintain stability/Regulate subcellular localization and transcriptional activity	(56, 100)
E-cadherin	Cytoplasmic domain	Block cell surface transport and reduce intercellular adhesion	(62)
Snail1	Ser112	Maintain stability and reduce E-cadherin mRNA expression	(69)
Vimentin	S34/S39/S49	Regulate homologous interactions, intermediate filament assembly	(70)
ZEB1	S555	Promote ferroptosis	(72)
SMAD4	T63	Inhibit interactions with GSK-3β and activate TGF-β	(71)
Cofilin	S108	Proper localization in invadopodia	(98)
Twist1	S31	Modulation of azacitidine (DAC) resistance in MDS/AML cells	(86)

Non-core molecules	O-GlcNAcylation sites	Function	Ref.

MORC2	T556	Activate SNAIL transcription	(80)
KAT5	S119	Activate TWIST1 transcription and enhance MMPs	(76)
KIF1A	/	promote OGT nuclear localization and nuclear β-catenin O-GlcNAcylation	(73)
UBR5	/	Degrade OGA and increase O-GlcNAc levels	(35)
FOXA1	T432/S441/S443	Suppress adhesion genes and reduce epithelial phenotype	(81)
STAT3	T717	Maintain stability, activate mesenchymal marker expression	(107)
CHOP	/	Promote CEBPB nucleation	(47)
RAF1	S227	Maintain protein stability and upregulate α-SMA, FN, and Vimentin expression	(108)

Over the last few decades, the fundamental roles of O-GlcNAcylation in EMT as well as its mechanisms for regulating EMT-related health and disease have undergone a surge in traction. O-GlcNAcylation is usually fluxed through the HBP, which integrates inputs from carbohydrates, lipids, amino acids and nucleotide pools into the crucial substrate UDP-GlcNAc. From the vantage point of glycosylation precursors, the first point is whether these source substances exert an influence on EMT through O-GlcNAcylation and the detailed regulatory mechanisms of O-GlcNAcylation in regulating EMT activation based on the impact of O-GlcNAc substrates on this modification. Of paramount importance are advances in our knowledge of how HBP or core upstream molecules that regulate the HBP enzymes during EMT have lagged, although proteomic modification advances have greatly improved our understanding of the rate-limiting enzyme of HBP in EMT network. Most importantly, the specific mechanisms underlying the substrate specificity of protein O-GlcNAcylation that involved in EMT remain poorly understood. This ubiquitous modification is cycled by only two enzymes: OGT, which cycles the O-GlcNAc moiety on proteins, and OGA, which cycles off from proteins. Nevertheless, this dynamic cycling activity exerts regulatory control over thousands of distinct protein substrates. An outstanding challenge in the field is to decipher how the pair of enzymes achieves selective recognition and modification of specific EMT transcription factors. Thus, we need a more comprehensive elucidation of not only how key O-GlcNAc-driven enzymes accurately identify and specifically modify EMT substrates spatio-temporally to develop EMT, but also the function of the entire molecules assigned to HBP during EMT signaling and physiological/pathological processes.

**Figure 5:**
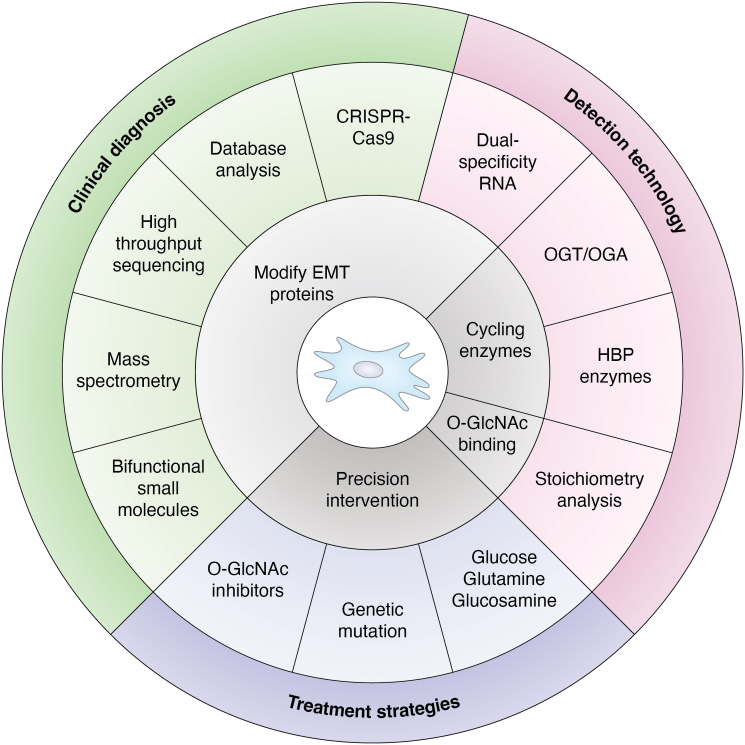
**Overview of emerging detection technologies, clinical diagnostics, and therapeutic strategies for O-GlcNAcylation in EMT.** Advancing O-GlcNAcylation research has shifted detection from western blotting to mass spectrometry and high-throughput sequencing. Clinical diagnostics leverages advanced technologies, from bioinformatics databases to genetic profiling, to precisely delineate pathological states and their mechanistic bases. These insights subsequently inform the development of targeted therapeutic strategies, which employ precision interventions such as protein engineering and enzyme modulation to rectify dysregulated metabolic pathways and correct disease-driving genetic alterations. Dysregulated O-GlcNAcylation acts as a central metabolic sensor, driving pathologies like cancer metastasis, neurodegeneration, and diabetic complications *via* EMT and other mechanisms. Future priorities include deciphering site-specific functional impacts across diseases, improving temporal resolution for detection, developing tissue-selective OGT/OGA modulators, and integrating multi-omics with functional validation to unlock the potential of precision medicine. EMT, epithelial-mesenchymal transition; OGA, O-GlcNAc hydrolase; OGT, O-GlcNAc transferase.

To date, a subset of molecules and O-GlcNAc modifications influencing EMT has been identified; however, it is plausible that a significant amount remains inadequately explored. As methodologies for investigating O-GlcNAcylation advance—exemplified by the emergence of innovative shark single-domain antibodies targeting OGT and dual-specificity RNA aptamers, characterizing O-GlcNAcylation will become considerably less challenging (102, 103). The function of EMT-TFs is significantly tissue-specific (SNAIL promotes metastasis in breast cancer, but not in pancreatic cancer (104)), and the mechanism may be related to tissue-specific co-regulators, but the exact molecular basis has not yet been elucidated. In the future, variations in the O-GlcNAc sites of EMT-TFs across various tumor types may be analyzed to determine if tissue-specific O-GlcNAcylation contributes to the functional heterogeneity of EMT-TFs by modifying their capacity to interact with tissue-specific co-factors. This approach may yield distinct therapeutic strategies for the targeted delivery of EMT-TFs to particular tumor types. The partial onset of EMT in malignancies is typically temporary and reversible, with its timing frequently aligned with changes in the tumor microenvironment and metabolic variations (23, 105, 106). O-GlcNAcylation, as a regulator of nutritional sensing *in vivo*, exhibits a swift response to rapid fluctuations. We propose that O-GlcNAcylation may function as a regulatory switch for controlling the activation and deactivation of EMT. Furthermore, we need to elucidate the molecular mechanism by which O-GlcNAcylation functions as a "metabolic timer" to sustain the dynamic equilibrium of EMT (Fig. 5).

In conclusion, O-GlcNAcylation, a crucial modification connecting metabolic status to cellular phenotypic shifts, presents potential as a significant advancement in elucidating the intricacies of EMT. As a dynamic translator converting nutrient flux into metastatic competence, this PTM illuminates previously unrecognized dimensions of cellular plasticity—revealing how metabolic dysregulation licenses transcriptional reprogramming and spatial reconfiguration during malignant progression. Deciphering its regulation of EMT master switches not only demystifies pathophysiological paradoxes in metastasis but also paves the way for a transformative therapeutic approach: precision targeting of O-GlcNAc circuitry offers unprecedented potential to disrupt the self-reinforcing metastasis cycle by simultaneously intercepting metabolic vulnerabilities, epigenetic dysregulation, and phenotypic plasticity.

## Conflict of interest

The authors declare that they have no conflicts of interest with the contents of this article.
